# Editorial: Biomaterials in cardiovascular research: Models, methods, and therapies

**DOI:** 10.3389/fbioe.2022.1116015

**Published:** 2023-01-04

**Authors:** Yingfei Xue, Payam Akhyari, Bin Duan, Hooi Hooi Ng, Zongmin Zhao

**Affiliations:** ^1^ Department of Surgery, Columbia University, New York, NY, United States; ^2^ Department of Cardiac Surgery and Research Group for Experimental Surgery, Medical Faculty, Heinrich Heine University, Düsseldorf, Germany; ^3^ Mary & Dick Holland Regenerative Medicine Program, Division of Cardiology, Department of Internal Medicine, University of Nebraska Medical Center, Omaha, NE, United States; ^4^ Kolling Institute, The University of Sydney, Sydney, NSW, Australia; ^5^ Department of Pharmaceutical Sciences, College of Pharmacy, University of Illinois at Chicago, Chicago, IL, United States

**Keywords:** cardiovasclar diseases, biomateials, medical implant, tissue engineering, cardiovascular device

The past decade has witnessed an exponential growth of novel biomaterial-based grafts, scaffolds, therapies, and diagnostic tools for cardiovascular systems. The advances in engineering biomaterials have resulted in biomedical implants with various functionalities tailored for the desired application. In the cardiovascular field, the application of biomaterials is often challenging but holds great promise. Heart and blood vessels are structurally complex tissues that are blood-contacting in nature and consistently under dynamic mechanical loading conditions. These biological and engineering challenges create unique opportunities for advanced biomaterial-based approaches to deepen our understanding of cell and tissue interaction and revolutionize novel therapeutics. To highlight the progress in the field, we launched this Research Topic to feature recent efforts in engineering novel biomaterials for cardiovascular repair and regeneration.

This Research Topic includes two review articles summarizing the clinical use of bovine jugular vein conduits and drug-eluting stents, two common cardiovascular implants. Li et al. first reviewed the clinical outcomes of current bovine jugular vein conduits and their common causes of failure. More importantly, the authors proposed strategies to modify and design the next-generation of bovine jugular vein conduits which have the potential to overcome the complications of current implants. Meanwhile, Hu and Jiang focused on the restenosis of cardiovascular stents. The authors provided a comprehensive summary of clinical reports, diagnostic tools, and common mechanisms of in-stent restenosis. It is important to note that the failure mechanisms and modification strategies not only apply to bovine jugular vein conduits or drug-eluting stents but a variety of other cardiovascular implants.

This Research Topic also includes four Original Research articles focusing on cardiovascular grafts, tissue engineering scaffolds, and injectable therapeutic. Qi et al. reported a novel tissue crosslinking strategy for bioprosthetic heart valve biomaterials. Using *in vitro* and *in vivo* models, the authors demonstrated that ribose-mediated crosslinking was advantageous over traditional glutaraldehyde-based crosslinking. Specifically, ribose crosslinking led to reduced calcification, immune responses, and degeneration of the extracellular matrix. Another common issue of cardiovascular implants is bacterial infection. Kloss et al. developed a novel antibacterial coating based on dalbavancin for small polymeric stent grafts. Using a mouse model of bacteria-induced implant infection, the authors showed the non-inferiority of dalbavancin coating when compared to the combination of rifampicin/minocycline coating in terms of anti-infective efficacy and inflammatory responses. Kitsara et al. innovated plasma-treated polyvinylidene fluoride scaffolds with improved wettability. The resulting scaffolds showed improved adhesion and maturation of primary cardiomyocytes *in vitro* and sustained the 28-day implantation on the surface of the mouse heart without eliciting major fibrosis. Finally, Yang et al. designed an injectable selenium-containing polymeric hydrogel to facilitate tissue repair post myocardial infarction. This antioxidative gel was shown to improve myocardial function, reduce fibrosis, and mitigate inflammatory responses in a mouse model of myocardial infarction.

We congratulate all authors who have contributed to this Research Topic on those exciting results. Those novel findings and opinions provide new insight into the enabling technologies of engineering biomaterials with unique functionalities and will inspire future studies to innovate and translate biomaterial-based cardiovascular therapies.

